# Biodiversity Evaluation and Preservation of Italian Stone Fruit Germplasm (Peach and Apricot) in Southern Italy

**DOI:** 10.3390/plants12061279

**Published:** 2023-03-11

**Authors:** Michele Antonio Savoia, Loredana Del Faro, Andrea Turco, Valentina Fanelli, Pasquale Venerito, Cinzia Montemurro, Wilma Sabetta

**Affiliations:** 1 Department of Soil, Plant and Food Sciences, University of Bari Aldo Moro, Via Amendola 165/A, 70126 Bari, Italy; 2 CRSFA-Centro Ricerca, Sperimentazione e Formazione in Agricoltura “Basile Caramia”, Via Cisternino 281, 70010 Locorotondo, Italy; 3 Spin Off Sinagri s.r.l., University of Bari Aldo Moro, Via Amendola 165/A, 70126 Bari, Italy; 4 Institute for Sustainable Plant Protection–Support Unit Bari, National Research Council (IPSP-CNR), Via Amendola 165/A, 70126 Bari, Italy; 5 Institute of Biosciences and BioResources, National Research Council (IBBR-CNR), Via Amendola 165/A, 70126 Bari, Italy

**Keywords:** *Prunus* species, local biodiversity, microsatellite transferability, genetic and phenotypic characterizations

## Abstract

The *Prunus* genus encompasses a group of economically important and closely related crops, sharing an essentially common genome and, thereby, a high level of conserved and transferable microsatellite (SSR) loci. In Southern Italy, many of the local and/or neglected varieties are abandoned and at risk of extinction due to the high degree of urbanization and agricultural intensification, despite their value as genetic resources for crop improvement. This research aimed to genetically and morphologically characterize the traditional apricot (*P. armenica*) and peach (*P. persica*) germplasms collected in old family orchards. Most of the official descriptor categories were scored, thus revealing a rather high level of phenotypic variation in both collections. Genetic data allowed the discovery of diversity masked by morphological traits. Genotyping in 15 and 18 SSRs, eight of which were transferable across both species, showed an average polymorphic informativeness (PIC) of 0.44 and 0.59 for apricot and peach, respectively, and a total of 70 and 144 alleles. A reliable identification of each genotype was achieved, and the presence of possible mislabeling and/or erroneous denominations was solved. These results are encouraging for the valorization of the still poorly explored Italian *Prunus* germplasm, with significant economic consequences for bioresource conservation and management.

## 1. Introduction

The peach (*Prunus persica* (L.) Batsch) and common apricot (*Prunus armenica* L.) belong to the *Rosaceae* family of the *Prunus* genus [1,2]. Together with other species, including plums and cherries, they are commonly known as “stone fruits” [1].

Originating in China and Central Asia [3], peaches and apricots are counted among the first examples of plant tree domestication, dating back to about 3–4000 years ago [4], and their spread in Europe was mainly due to the Romans [5,6,7]. Over the centuries, the temperate climate and the different orographic conditions have favored the spread of peach and apricot in Italy, too, particularly in the Apulia region, where the cultivation of these two species still plays a relevant role in the regional economy, with an average annual production of peaches and apricots equal to 10 and 2 million quintals, respectively [8].

Similarly, to other tree fruit species, the peach and apricot have also caught the attention of farmers, consumers and scientists in the last twenty years, thanks to their richness in metabolites with nutraceutical properties, such as carotenoids, anthocyanins, phenolic acids and flavonoids. These secondary metabolites are well known to generally improve food quality and exert beneficial effects on human health, such as in the prevention of tumors and age-related diseases [9,10,11]. Apricots have an important nutritional and health profile, containing high amounts of sugars, fibers, proteins, minerals and vitamins [12]. Peaches, with their high content of phenolic compounds and vitamins (e.g., ascorbic acid and vitamin C), might exert a strong antioxidant activity in mammalian cells [13]. However, despite being one of the most consumed fruits in the world, the peach market has recently suffered a sharp collapse due to a progressive reduction of fruit quality and organoleptic properties that have displeased consumer expectations [14]. Intensive and monovarietal agriculture, inadequate agronomic practices, the harvest of unripe fruits and the presence of post-harvest structural damages are some of the causes of reduced consumption of peaches [15]. In addition, other factors such as severe winter frosts in recent years, the scarce availability of cultivation areas and the action of aggressive pathogens [16,17,18] have contributed to causing a decrease in the production and marketing of both peaches and apricots [19].

Together, these issues have increased the interest in traditional and local varieties and the global importance of safeguarding and protecting biodiversity. In fact, ancient and rare plant germplasms could represent a new source of agronomically important traits and genes, which could face climate changes and the onset of new pathogens. In recent years, different integrated projects have been financed with European funds at a regional level, aiming for the recovery and protection of the existing biodiversity and the germplasms of several Apulian tree crop species [20,21,22], including peach and apricot. Several actions have been carried out in this regard: historical investigation and cataloging, morphological and genetic characterization, phytosanitary assessment, as well as the ex situ conservation of local germplasm.

The genetic structure of peach and apricot populations is strongly related to the reproductive system that characterizes these species. The peach is a homozygous and predominantly self-pollinating species with a high degree of auto-compatibility [23]. In addition, most peach cultivars are derived from a restricted parental material, thus causing a narrow genetic base and reduced genetic diversity [1]. This low heterogeneity makes it very difficult to differentiate genotypes and determine the genetic diversity of peach germplasm. On the contrary, the apricot has a cross-pollination reproductive system with a higher genetic variability that is dependent on a moderate gene flow and the level of auto-incompatibility of the species [24].

Several studies have analyzed the morphological and genetic diversity of apricot and peach collections, although few have directly compared these two closely related species. Recent works have underlined the great potential of some apricot accessions to be directly cultivated or employed in breeding programs thanks to their high level of phenotypic [25] and genetic [26,27] variability. Similarly, some studies about peaches mainly aimed to study the genetic variation among cultivars and to preserve the existing variability [28,29,30,31]. In this research, we aimed to dissect the morphological and genetic biodiversity of two Apulian ex situ collections comprising 57 apricot and 59 peach cultivars, respectively. The morphological evaluation was based on the measurement of the most important GIBA and UPOV descriptors, while the genetic study made use of SSR molecular markers. Since the DNA sequences flanking SSRs are preserved between different taxa [32] and the transferability of SSR markers between species belonging to the same genus has been demonstrated in-depth [33,34,35], some microsatellite markers, previously and successfully applied to almond species (*Prunus dulcis* Mill. D.A., syn. *Prunus amygadulus* Batsch, syn. *Amygdalus communis* L.) [36,37,38], have been used here.

## 2. Results and Discussion

### 2.1. Cross-Transferable SSR Amplification and Genetic Variability Analysis

Microsatellite markers (SSRs) are an invaluable tool for germplasm characterization thanks to some advantageous properties, such as their codominance, multi-allelism, high polymorphism rate, robustness and analytical simplicity. Moreover, the extensive synteny existing among the *Prunus* species [39] makes possible the transferability of microsatellite markers across species belonging to the *Prunus* genus [40,41], i.e., their ability to amplify a single or few loci in different *Prunus* species and result in polymorphism. Thus, SSRs have been widely applied for genotyping cultivars, especially in traditional mixed orchards, often characterized by the simultaneous cultivation of several related species.

In this study, 15 and 18 SSRs were used to assess the genetic diversity of apricot and peach populations, respectively; among those, 8 SSRs have been proven to be transferable across the two species, providing successful amplification profiles. Most loci were highly polymorphic and informative, and the allele lengths were consistent with the literature. Considering the whole collection of apricots and peaches analyzed with eight common microsatellites, a total of 89 alleles were detected, ranging from 8 for the CPPCT033 marker to 14 for the BPPCT025 marker (Table 1). Moreover, 18 alleles were identified as unique and 28 as rare, with allele frequencies less than 1 and 5%, respectively. The diversity parameters in the whole collection were generally quite high, with mean values of the observed heterozygosity (Ho) and the polymorphism information content (PIC) of 0.408 and 0.748, respectively. Markers BPPCT001, BPPCT007 and UDP98412 resulted the most polymorphic ones, while CPPCT033 provided the less informative SSR with the lowest values for all the genetic indexes and the fewest number of distinguishable genotypes (Table 1).

When comparing these results within the single species, some differences among the SSR features were found (Table 2). For example, the BPPCT001, BPPCT025 and CPDCT045 loci resulted in less information for the apricot germplasm than the whole collection, with lower Na, PIC and Ho numbers. Similarly, in the peach collection, UDP98409 was the marker with the lowest values of all the genetic indexes compared to those obtained by the simultaneous analyses of both species. Furthermore, the genetic variability detected within the single species was moderately high, despite the restricted geographical area of the survey (Apulia region, Southern Italy), with the results comparable to similar studies, as detailed in the following paragraphs.

In particular, the genetic profiles of 65 apricot genotypes by 15 SSRs allowed a total of 70 alleles to be detected, 20 of which were rare and found in 30 genotypes, while 8 were unique and found in 5 genotypes. The highest numbers of rare and unique alleles were recorded for marker CPDCT025 and for markers UDP98409 and CPPCT006, respectively. The Na mean value in the Apulian apricot collection was five alleles per locus (Table 2), which is comparable with what was reported by [24] for a Siberian apricot collection (4.5). Higher Na values have been observed in other studies about Chinese wild apricot germplasm (23) [42], landraces and domesticated cultivars (16.7) [43], native apricot accessions from Europe, China and Central Asia (7.6) [32] and other *Prunus* genotypes (15.14) [44]. According to other reports, the number of analyzed loci and the population size included in the study might affect the estimates of genetic diversity [45,46].

Generally, the discrimination power of most microsatellites ranged from 0.06 to 0.73, with a mean value of 0.44 (Table 2), which is less than the 0.67 reported by [46] but higher than what was reported by [47] and [26]. Closer values for Ho and He were recorded, with mean values of 0.45 and 0.49, respectively, which were consistent with the literature [24,26,32,48,49]. The fixation index (F, mean value 0.09) confirmed a good level of heterozygosity in this collection and a moderate level of gene flow, probably due to the predominantly cross-pollinating and self-incompatible reproductive system of the apricot [24]. The Shannon’s Information Index confirmed a significant level of genetic diversity in the Apulian apricot population (Table 2), with it being higher than what was found in Indian apricot cultivars (0.63) by [26] and in Chinese wild apricot (0.45) by [42] but lower than what was found in Southern Italian neglected germplasm (1.36) [46], in common Chinese apricots (2.06) [44] and in a worldwide germplasm collection of *P. armenica* (2.5) [50].

The analysis of the peach collection that was constituted of 64 genotypes (59 local plus 5 commercial genotypes) allowed the detection of a total of 144 alleles across 18 microsatellite loci, with an average of 8 Na and 3 Ne per locus (Table 2). This great difference between Na and Ne implied that a large portion of these alleles had a frequency of less than 5%. In fact, rare and unique alleles were, respectively, detected in 40 and 15 genotypes for most of the considered loci, but the highest numbers were found for BPPCT015 and BPPCT025. This finding is quite common in numerous studies on peach genetic diversity since high percentages of rare and unique alleles are often reported [1,6,24,29,51]. In our study, the “Giallo di Vico” and “Pesco Maschio” cultivars resulted in the richest rare and unique alleles. The PIC value averaged at 0.59, in accordance with [29], thus revealing a moderate degree of polymorphism for all loci. A mean Ho of 0.373 was observed, while the He was 0.628; consequently, the F values were positive for all loci with a mean of 0.37. Finally, the average I value (1.343) confirmed the discrete level of genetic diversity in the studied collection (Table 2).

The genetic results of this investigation are generally comparable to those obtained in similar studies about peach germplasm characterization [6,28,29,30,47], even if some considerations, such as the collection size along with the number and the polymorphism levels of the used SSRs, may be behind the most relevant differences between studies. For example, higher Na and PIC values are typically reported when a conspicuous number of SSR markers is applied, and the analyzed germplasm is quite heterogeneous, including local and foreign genotypes or modern and wild accessions from different sampling sites [28,51,52]. Despite the tendency of the peach to self-pollinate [23], its propagation by grafting generally allows it to preserve the selected cultivars as well as the heterozygosity [51]; thus, the moderate variability found in the Apulian peach germplasm brings to light the richness and the relevance of traditional and local genotypes.

In order to investigate the putative cases of synonymy in both collections, an LRM (pairwise relatedness) analysis was performed by fixing 0.50 as the maximum value for identical genetic profiles. For apricots, several cases of complete genetic identity were observed (Table 3). With regard to peaches, only one case of a strong genetic relationship was observed for the cultivars “Persichine_Apritune” and “Aprituna_2”, highlighting a case of synonymy not reported in previous studies (Table 3).

### 2.2. Genetic and Phenotypic Characterization of Apricot Collection

The relationships among the apricot genotypes were elucidated by a cluster analysis of the genetic and morphological distance matrices.

**Figure 1:**
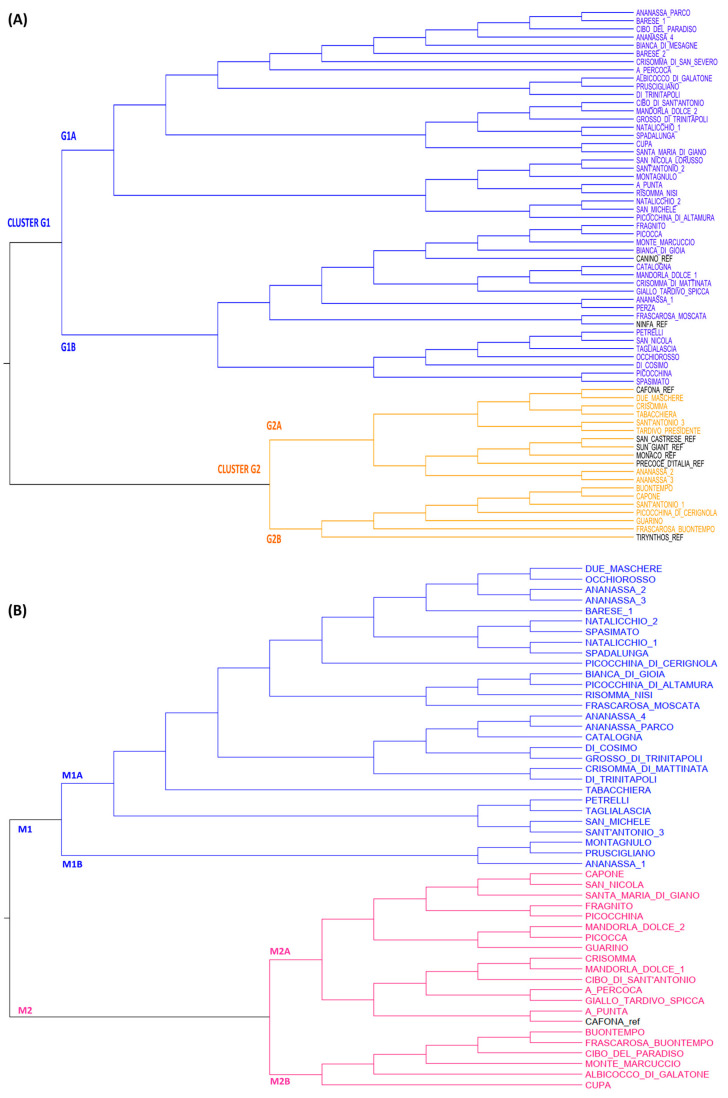
Dendrogram (**A**) of the Apulian apricot collection based on genetic distance from 15 SSR data and (**B**) of a subset of the collection based on 33 phenotypic traits. Genotype names are colored according to the main cluster they belong to. Reference cultivars are reported in black.

The phylogenetic tree divided the Apulian apricot collection into two main clusters (Figure 1A): cluster G1, the largest one, which contains most of the analyzed genotypes (46), and cluster G2, which includes the remaining genotypes (19). Both clusters were further divided into two sub-groups each, named G1A, G1B and G2A, G2B. The clusterization of some reference cultivars was in line with the results of other research, which confirmed the high genetic distance between “Canino” and “Tirynthos” [27] and the close genetic relation between “San_Castrese” and “Cafona” [53].

Phylogenetic clustering confirmed most of the LRM results since the cultivars with high genetic similarity were located in the same branch of the tree, such as in the case of the genotypes belonging to the groups named “Ananassa” and “Picocca”. In addition, erroneous cases of homonymy have been solved since cultivars with the same name but clearly with different genetic profiles were distinguished. For example, the cultivars “Barese_1” and “Barese_2”, or “Mandorla_Dolce_1” and “Mandorla_Dolce_2” or “Natalicchio_1” and “Natalicchio_2” resulted genetically distinguishable from each other. Similarly, the group of “Sant’Antonio” cultivars showed high molecular heterogeneity (Figure 1A).

**Figure 2:**
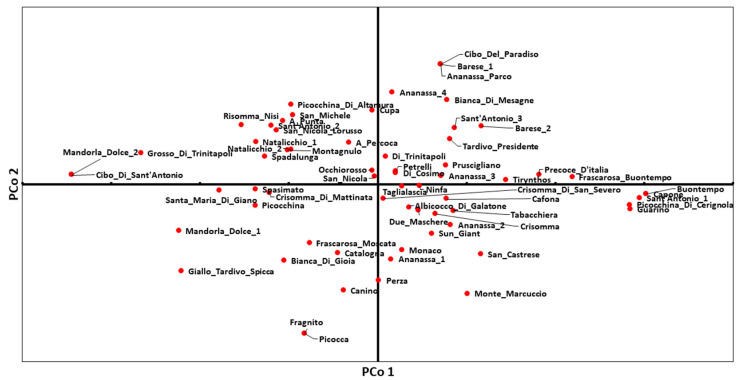
Principal Coordinate Analysis (PCoA) of 65 Apulian apricot genotypes.

The principal coordinate analysis (PCoA) was not as informative as expected because no well-defined clusters were clearly identified. However, the genetic distance for the reference cultivars and most cultivars with LRM values of 0.5 was respected. A slight discrepancy with the LRM results and the phylogenetic clustering was observed in some cases, such as those for the couples “Natalicchio_2” and “San_Michele”, “Petrelli” and “San_Nicola”, “Picocchina” and “Spasimato”, and “Cafona” and “Due_maschere” that did not perfectly overlap in the PCoA plot (Figure 2).

A subset of the apricot collection (50 samples) was subjected to a phenotypic characterization by means of 33 morphological and phenological traits (Table S1). The phenotypic clustering did not always mirror the genetic classification (Figure 1B) since several cases of genetically very close cultivars resulted remarkably differently from a morphological point of view. For example, the phenotypes of cultivars “Spasimato” and “Picocchina”, “Due_Maschere” and “Cafona”, and “Petrelli” and “San_Nicola” substantially differed from one another, despite their LRM values of 0.5. Thus, each component of those couples was separated into two main morphological clusters, M1 and M2. The cultivar “Cibo_del_Paradiso”, which was genetically closer to the “Ananassa” group (LRM = 0.5), resulted in being morphologically different and fell alone in the M2 cluster. On the other hand, some quite genetically distant cultivars belonging to different G clusters (such as “Picocchina_di_Altamura” and “Picocchina_di_Cerignola”) showed high phenotypic similarity and lay closer on the same M1A branch. These controversial results between the genetic and the phenotypic classification could be due to not only the biological differences among the cultivars in relation to their origin, mutation level, inter-crossing and evolutionary changes but also the use of not always comparable methodological parameters, as also suggested by [1], who obtained similar results. Consistent with studies for other tree crops [54,55], our results suggest that the estimation of apricot variability exclusively based on morphological traits can misrepresent the level of diversity.

**Figure 3:**
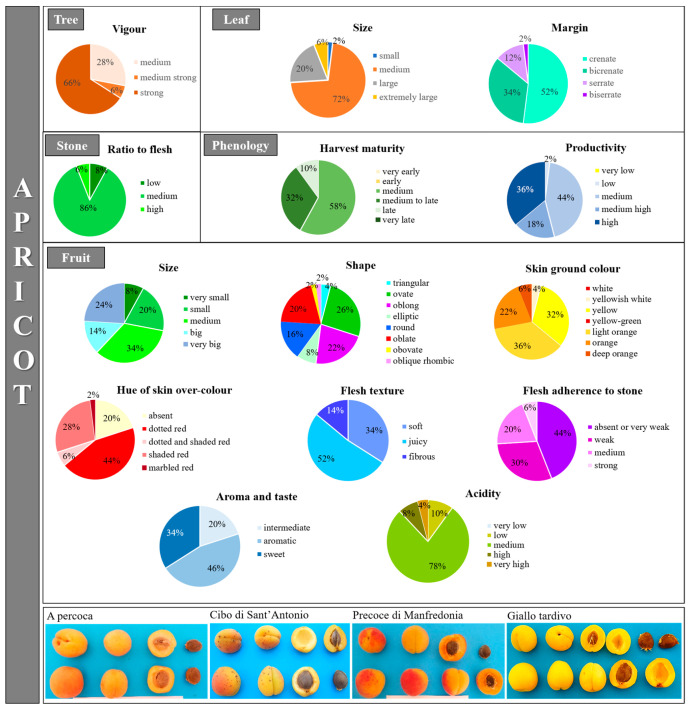
Graphical representation of apricot morphological and phenological categories for some of the considered traits (for all traits, see Figure S1). For each category, the percentage of scored genotypes in the collection is reported. At the bottom, pictures representing the biological variation in some fruit and stone characteristics are shown.

The considered phenotypic traits resulted polymorphic with at least two different categories each (Figure 3 and Figure S1). In general, a huge variation was observed among the cultivars, mostly concerning the fruit’s quality-related attributes, leaf morphology and some phenological traits, as also reported by other authors [25,49,54,55]. Among the most variable traits were fruit size, shape and acidity, the skin ground color and hue of the skin over-color, the flesh color and adherence to the stone. For those traits, all the possible categories listed in the UPOV and GIBA guidelines [56,57] were present, considering the scored phenotypes and their distribution. For example, very small to very big fruits were found with shapes ranging from triangular to oblong, round, rhombic, etc. The skin ground color of most fruits (36%) was light orange, although many yellow (32%) and orange (22%) fruits were also present. Most apricot fruits were aromatic (46%) with medium acidity levels (78%) and a very weak adherence of flesh to the stone (44%). On the contrary, despite the numerous listed categories, the tree habit proved to be the less variable trait with a single predominant phenotype (100% spreading). In general, this Apulian apricot collection resulted moderately (44%) to highly (36%) productive (Figure 3 and Figure S1). The traits associated with the external appearance of the fruit, including the fruit dimensions, color and shape, are generally relevant for consumers since they have a direct impact on the sales and acceptance for both the fresh and processed markets. Furthermore, these traits are also important for packaging and transportation. Thereby, the apricot genotypes with the highest values regarding the most important fruit quality traits can be treated as potential superior accessions to be directly used for cultivation or in breeding programmes.

### 2.3. Genetic and Phenotypic Characterization of Peach Collection

**Figure 4:**
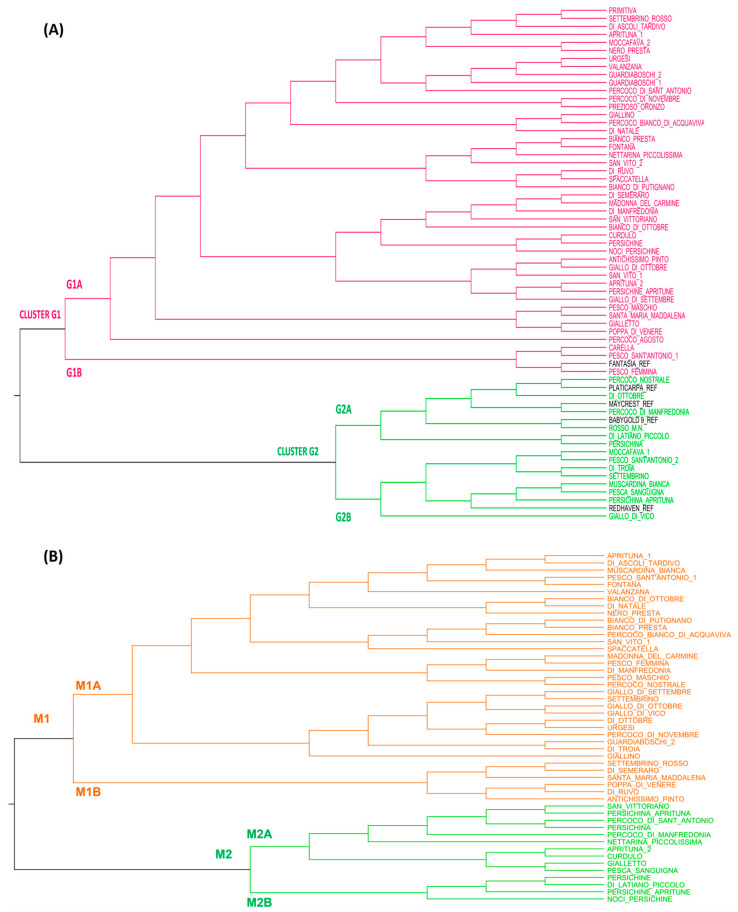
Dendrogram (**A**) of the Apulian peach collection based on genetic distance from 18 SSR data and (**B**) of a subset of the collection based on 36 phenotypic traits. Genotype names are colored according to the main cluster they belong to. Reference cultivars are reported in black.

The phylogenetic tree divided the Apulian peach collection into two main clusters: G1 and G2, which, in turn, included at least two sub-clusters each, called G1A and G1B, and G2A and G2B (Figure 4A). Most genotypes (46) were included in cluster G1, while 18 genotypes were grouped in cluster G2. Except for one, all commercial cultivars here used as references were included in cluster G2. The genetic distance between the references “Fantasia”, “Maycrest”, and “Redhaven”, found by [58], was confirmed but partially contrasted with the study of [31].

The clustering of Apulian local peaches supported the LRM results because cultivars with high genetic similarity were found to belong to the same tree branch (Figure 4A). The close genetic distance between “Persichine_Apritune” and “Aprituna_2” confirmed that they share the same genetic profile (LRM = 0.5). Strong genetic relationships have also been confirmed for couples with 0.42 < LRM < 0.49. Despite a low LRM value (0.08) between the cultivars “Guardiaboschi_1” and “Guardiaboschi_2”, a close genetic distance between them emerged and thereby needs further clarification. Similar to the apricot, the clustering analysis of peaches allowed for some cases of homonymy or incorrect denomination to be identified. For example, the components of the couples “Moccafava_1” and “Moccafava_2” and “Pesco_Sant’Antonio_1” and “Pesco_Sant’Antonio_2” were separated in the two main clusters, although they have similar names. The genotypes typically classified as “Percoco” were found to be quite distant from each other in the phylogenetic tree and, therefore, genetically heterogeneous (Figure 4A).

**Figure 5:**
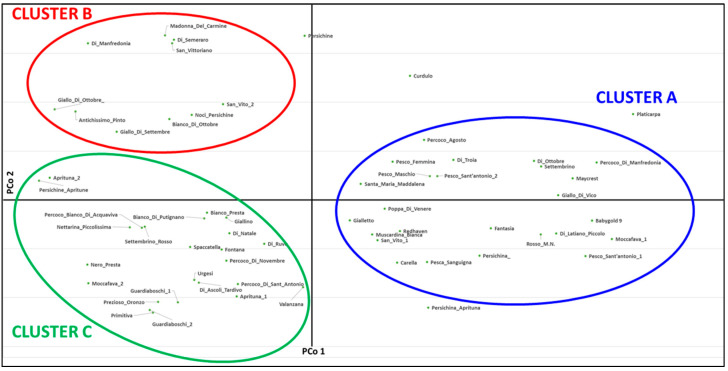
Principal Coordinate Analysis (PCoA) of 64 peach genotypes. The three clusters are marked with different colors (blue for cluster A, red for cluster B and green for cluster C).

The PCoA plot was consistent with the genetic clustering tree, confirming the allelic similarity of the cultivars with high LRM values and the resolution of wrong homonymous cases. The first two components explained 16.63% of the total genetic variation (9.04 and 7.59%, respectively). According to that, the cultivars were divided into three main groups: A, B and C (Figure 5). In detail, cluster A mostly corresponded to cluster G2 of the phylogenetic tree, while clusters B and C contained genotypes that were included in cluster G1. Only four genotypes (“Persichine”, “Persichina_Aprituna”, “Curdulo” and the reference “Platicarpa”) were excluded from the PCoA clusters, thus highlighting their high genetic diversity with respect to the entire peach collection.

The phenotypic variation in a subset of peach genotypes (49) was assessed by 36 morphological and phenological traits (Table S1). Again, the presence of two main groups (M1 and M2) with different sizes and two sub-clusters each was confirmed (Figure 4B). However, in this case, some genetic classification discrepancies emerged because some cultivars could not be grouped in the same clade as expected. In fact, cultivars that were genetically very close were placed under different phenotypic clusters, and/or members of different morphological groups showed high LRM values, thus causing the two dendrograms not to overlap perfectly. For example, the cultivars from the group “Persichine/Persichina”, which belonged to different genotypic clusters, fell into the same morphological M2 cluster, thus potentially explaining the attribution of similar names. The group of “Percoco” genotypes resulted in a distribution among different branches of the morphological tree. On the contrary, although the cultivars “Aprituna_2” and “Persichine_Apritune” were genetically identical (LRM = 0.5), they showed some morphological differences that caused them to fall into different sub-clusters (M2A and M2B). For peaches, too, many factors could have contributed to the complication of the genetic background of this species and thus caused differences between the classification methods based on genetic and morphological information.

**Figure 6:**
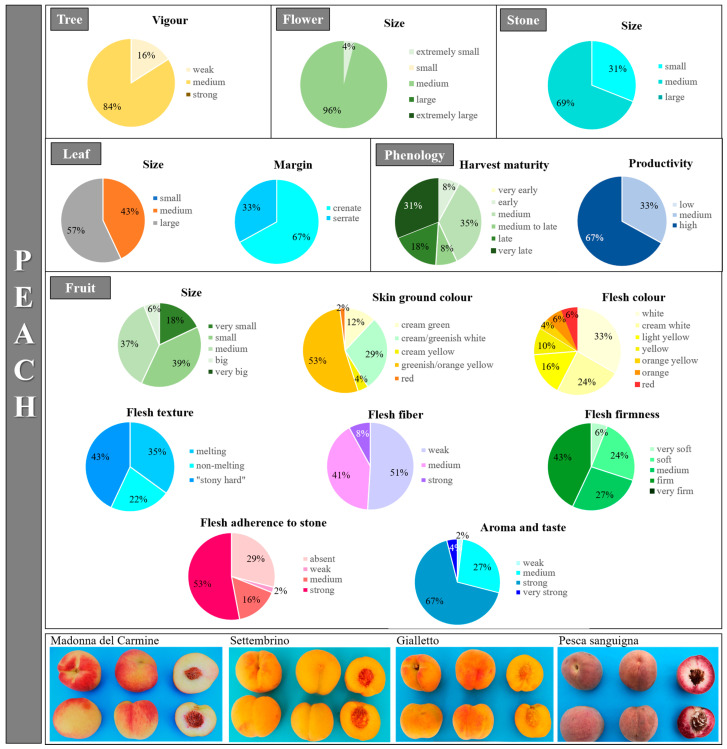
Graphical representation of peach morphological and phenological categories for some of the considered traits (for all traits, see Figure S2). For each category, the percentage of scored genotypes in the collection is reported. At the bottom, pictures representing the biological variation in some fruit and stone characteristics are shown.

Phenotypic traits displaying substantial differences in their range of variation were related to phenology and fruits, as reported by other studies [59,60] (Figure 6 and Figure S2), while a low variability was found only for the traits related to the tree since most of the scored phenotypes showed a spreading habit (96%) and medium vigor (84%). The harvest of maturity strongly varied among the genotypes, ranging from early (8%) to very late (31%), although the majority (36%) of the varieties fell into the medium to late category. Fruits of about half of the collection (51%) were found to be slightly resistant to harvest and post-harvest handling, while the remaining part resulted in medium (29%) or high (18%) resistance. Many of the fruit-related categories officially reported in the peach descriptor list were represented in the collection. For instance, the fruit size ranged from very small (18%) to small (39%), medium (37%) and big (6%); a wide variability of the skin ground color was observed, too, varying from cream-green to red. The hue and pattern of skin over-color were also highly represented across all the possible categories. The flesh morphological features, such as color, firmness and adherence to the stone, greatly varied in the collection. Among all the present categories, the most common phenotypes were the white flesh color (33%), firm flesh (43%), “stony hard” texture (43%), strong fiber (51%) and strong flesh adherence to the stone (53%). Finally, most of the analyzed Apulian peach fruits had a strong aroma and taste (67%).

Similar to apricots, the characteristics of peaches can attract potential consumers, too, whose acceptance and satisfaction boost the market value of the fruits themself. Additionally, the fruit’s size and shape can also affect post-harvest handling. Thereby, considering consumer trends, global climate warming and environmental concerns, stakeholders generally prioritize some important traits, such as the fruit’s size, shape and color, texture and sweetness, fruit developmental stage and harvest time.

## 3. Materials and Methods

### 3.1. Plant Material

This study was conducted on 57 apricot and 59 peach genotypes collected from different orchards spread in the Apulia region (Southern Italy). All genotypes belonged to the ex situ collection of the CRSFA Institute (Centro di Ricerca, Sperimentazione e Formazione in Agricoltura “Basile Caramia”), located in Locorotondo (Bari). They were considered ancient, rare or neglected varieties to be preserved from the risk of extinction. In addition, some commercial apricot (8) and peach (5) varieties provided by CRSFA were included in the analysis and used as references.

### 3.2. DNA Extraction and SSR Analysis

Genomic DNA was extracted from powdered young leaves, according to [61]. DNA concentrations and purification parameters were assessed by the use of a Nanodrop ND-1000 Spectrophotometer (Thermo Fisher Scientific, Waltham, MA, USA) and 0.8% agarose gel electrophoresis (Bio-Rad Laboratories, Hercules, CA, USA). All concentrations were normalized to 50 ng/μL with sterile water. The genetic variability of the peach and apricot collections was assessed using two sets of nuclear SSRs, specifically 15 for apricots and 18 for peaches (Table S2).

The amplification reactions were performed, as in [61]. All forward primers were labeled with one of the following dyes: 6FAM, NED, VIC or PET. The same amplification protocol was applied for all used SSRs, comprising an initial denaturation at 95 °C for 5 min, with 35 cycles of 95 °C for 45 s, followed by annealing at a single temperature of 56 °C for 45 s and 72 °C for 45 s, and a final extension at 72 °C for 8 min. The PCR products were prepared for automatic capillary electrophoresis, which was run as described in [61] and genotyped by the GeneMapper v.5.0 software (Applied Biosystems, Foster City, CA, USA).

### 3.3. Genetic Diversity

The following statistical parameters were calculated for each SSR marker by the use of GenALEX v. 6.51b2 software (http://biology-assets.anu.edu.au/GenAlEx, accessed on 6 June 2022) [62]: Ne, I [63], Ho, He and F [64]. Moreover, the number of unique and rare alleles (frequency <1% and <5%, respectively) for a single locus and single genotype within the two collections was estimated. The allelic similarity between the examined cultivars was analyzed, and the cases of synonymy and homonymy were discovered through marker-based relatedness (LRM) [65]. The PIC values were calculated using Cervus v. 2.0 software.

In order to evaluate the genetic relationship between the varieties, both in the individual collections and in the group as a whole, the simple matching dissimilarity index was applied. A weighted neighbor-joining tree [66] was computed using the Dissimilarity Analysis and Representation for Windows (Darwin5) software, version 6.0.010 (http://darwin.ci-rad.fr, accessed on 7 June 2022). The robustness of branches was tested using 1000 bootstraps [67]. Moreover, a similarity/dissimilarity matrix was elaborated by a principal coordinates analysis (PCoA) [68] using the GenALEX software.

### 3.4. Morphological Characterization

Several phenotypic traits were analyzed for each genotype over two consecutive years. Specifically, 30 and 32 morphological traits and 3 and 4 phenological traits were coded for a subset of apricot and peach genotypes, respectively (Table S1), according to the list of UPOV and GIBA descriptors [56,57]. All morphological data were converted in a data matrix to obtain a neighbor-joining dendrogram by the use of Darwin software with 10,000 bootstrap replications [67].

## 4. Conclusions

In this study, an investigation of the genetic and phenotypic diversity of apricot and peach germplasms in the Apulian region (Southern Italy) has been illustrated. The relevance of the combined dual approach is highlighted for the accurate and proper identification of genotypes, especially those neglected or never studied before. A rather extensive variation has been found among the cultivars of the examined species with both approaches. All genotypes were clearly identified and distinguished, and a cluster analysis enabled the establishment of similar groups of genotypes. The possibility of separating morphologically related accessions by applying genetic tools has also been demonstrated, which is an important issue, particularly for local germplasms subject to possible genetic erosion and worthy of being preserved. Thus, these results have underlined how the local genotypes with interesting values, in terms of fruit quality traits and/or productivity, can have the potential to be used for direct cultivation as well as in breeding programs, especially in the current scenario of severe climate change.

## Figures and Tables

**Table 1:** Summary of the main genetic indexes of 8 SSR markers used for analyzing the whole collection of apricots and peaches: locus name, number of detected genotypes (N), number of detected (Na) alleles, observed (Ho) and expected (He) heterozygosity, and polymorphism information content (PIC) are reported.

SSR Locus	N	Na	Ho	He	PIC
BPPCT001	124	13	0.419	0.831	0.812
BPPCT007	123	10	0.528	0.824	0.802
BPPCT025	128	14	0.406	0.790	0.763
CPDCT045	123	9	0.325	0.711	0.667
CPPCT006	126	10	0.540	0.786	0.759
CPPCT033	115	8	0.148	0.649	0.585
UDP98409	129	13	0.457	0.773	0.749
UDP98412	127	12	0.441	0.863	0.848
*Total*	*129*	*89*	*-*	*-*	*-*
*Mean*	*-*	*11*	*0.408*	*0.778*	*0.748*

**Table 2:** Genetic parameters were calculated by SSR analysis for the analyzed *Prunus* species. Locus name, allele size range (base-pair, bp), number of detected (Na) and effective (Ne) alleles, observed (Ho) and expected (He) heterozygosity, fixation index (F), Shannon’s index (I) and the PIC value are reported. Successful cross-transferable SSRs are highlighted.

Crop Name	SSR Locus	Allele Size	Na	Ne	I	Ho	He	F	PIC
**Apricot**	BPPCT001	111–117	2	2	0.662	0.350	0.469	0.25	0.36
	BPPCT007	135–167	5	3	1.088	0.594	0.609	0.03	0.54
	BPPCT010	114–120	2	2	0.615	0.391	0.424	0.08	0.33
	BPPCT014	184–186	2	1	0.148	0.068	0.065	−0.04	0.06
	BPPCT025	145–157	4	2	0.810	0.563	0.478	−0.18	0.41
	CPDCT025	196–202	9	2	1.154	0.477	0.501	0.05	0.48
	CPDCT045	128–134	3	1	0.377	0.153	0.198	0.23	0.18
	CPPCT006	184–202	9	4	1.654	0.774	0.747	−0.04	0.72
	CPPCT033	137–141	3	1	0.459	0.151	0.235	0.36	0.22
	CPSCT012	142–174	4	1	0.547	0.246	0.283	0.13	0.26
	CPSCT018	139–147	4	2	1.066	0.590	0.582	−0.01	0.53
	Pchgms1	156–172	5	3	1.222	0.651	0.665	0.02	0.61
	UDP96003	91–109	4	2	0.946	0.443	0.555	0.20	0.48
	UDP98409	134–164	9	4	1.732	0.662	0.765	0.14	0.73
	UDP98412	82–114	5	3	1.357	0.635	0.702	0.10	0.65
	*Mean*	*-*	*5*	*2*	*0.922*	*0.450*	*0.485*	*0.09*	*0.44*
**Peach**	BPPCT001	131–161	11	6	1.963	0.492	0.829	0.41	0.81
	BPPCT007	127–149	7	3	1.488	0.458	0.710	0.36	0.68
	BPPCT015	150–236	18	6	2.196	0.508	0.830	0.39	0.82
	BPPCT017	132–178	11	2	1.097	0.385	0.474	0.19	0.45
	BPPCT025	173–195	10	3	1.593	0.262	0.690	0.62	0.66
	BPPCT038	124–154	8	2	1.209	0.484	0.542	0.11	0.52
	CPDCT045	138–154	6	3	1.133	0.492	0.617	0.20	0.55
	CPPCT006	174–192	7	3	1.334	0.323	0.675	0.52	0.63
	CPPCT022	248–294	7	4	1.570	0.415	0.753	0.45	0.72
	CPPCT033	143–157	5	2	0.705	0.143	0.348	0.59	0.32
	CPPCT044	149–191	10	4	1.742	0.323	0.758	0.57	0.73
	CPSCT012	154–166	7	2	0.999	0.406	0.484	0.16	0.45
	EPPCU5176	118–128	5	2	0.866	0.354	0.452	0.22	0.41
	UDP96005	152–172	8	4	1.561	0.354	0.748	0.53	0.71
	UDP96008	134–164	6	2	1.119	0.492	0.546	0.10	0.51
	UDP98022	124–136	5	4	1.427	0.308	0.743	0.59	0.70
	UDP98409	120–152	6	1	0.676	0.262	0.328	0.20	0.30
	UDP98412	106–130	7	4	1.502	0.262	0.748	0.65	0.71
	*Mean*	*-*	*8*	*3*	*1.343*	*0.373*	*0.626*	*0.38*	*0.59*

**Table 3:** List of pairwise relatedness based on the LRM estimator.

Crop Name	Genotypes with LRM = 0.50	
**Apricot**	Ananassa_2	Ananassa_3
	Ananassa_4	Ananassa_Parco
	Ananassa_4	Barese_1
	Ananassa_4	Cibo_Del_Paradiso
	Buontempo	Capone
	Buontempo	Guarino
	Buontempo	Picocchina_Di_Cerignola
	Cibo_Di_Sant’Antonio	Grosso_Di_Trinitapoli
	Cibo_Di_Sant’Antonio	Mandorla_Dolce_2
	Cafona_ref	Due_Maschere
	Fragnito	Picocca
	Natalicchio_1	Spadalunga
	Natalicchio_2	San_Michele
	Occhiorosso	San_Nicola
	Petrelli	Taglialascia
	Picocchina	Spasimato
	Montagnulo	San_Nicola_Lorusso
	Montagnulo	Sant’Antonio_2
	**Genotypes with 0.40 < LRM < 0.50**	
	A_Punta	Sant’Antonio_2
	A_Punta	Risomma_Nisi
	A_Punta	San_Nicola_Lorusso
	Buontempo	Sant’Antonio_1
	Buontempo	Frascarosa_Buontempo
	Capone	Sant’Antonio_1
	Capone	Frascarosa_Buontempo
	Frascarosa_Buontempo	Guarino
	Frascarosa_Buontempo	Picocchina_Di_Cerignola
	Guarino	Sant’Antonio_1
	Montagnulo	San_Michele
	Montagnulo	Natalicchio_2
	Natalicchio_2	Sant’Antonio_2
	Natalicchio_2	San_Nicola_Lorusso
	Natalicchio_2	Risomma_Nisi
	Occhiorosso	Petrelli
	Occhiorosso	Taglialascia
	Petrelli	San_Nicola
	Picocchina_Di_Cerignola	Sant’Antonio_1
	Risomma_Nisi	San_Michele
	San_Michele	Sant’Antonio_2
	San_Michele	San_Nicola_Lorusso
	San_Nicola	Taglialascia
	Sant’Antonio_3	Tardivo_Presidente
**Peach**	**Genotypes with LRM = 0.50**	
	Persichine_Apritune	Aprituna_2
	**Genotypes with 0.40 < LRM < 0.50**	
	Moccafava_2	Nero_Presta
	Giallo_Di_Ottobre_	Antichissimo_Pinto

## Data Availability

All data of molecular and morphological characterizations have been collected in an integrated database with the regional GIS portal, accessible on the Apulia region website (www.psr.regione.puglia.it, accessed on 28 September 2022) on request. Historical information and detailed pictures of apricot and peach genotypes are reported in the “Atlante dei frutti antichi di Puglia”, available at the link: http://www.fruttiantichipuglia.it/atlante-dei-frutti-antichi-di-puglia/, accessed on 28 September 2022.

## Supplementary Materials

The following supporting information can be downloaded at: https://www.mdpi.com/article/10.3390/plants12061279/s1; Table S1: List of morphological and phenological traits recorded in apricot and peach collections.; Table S2: List of microsatellites used to analyze apricot and peach collections. Locus type, species of origin (Prunus spp.), repeat motif and annealing temperature (Ta in °C) are specified; Figure S1: Graphical representation of apricot morphological and phenological categories detected for all the considered traits. For each category, the percentage of scored genotypes in the collection is reported; Figure S2: Graphical representation of peach morphological and phenological categories detected for all the considered traits. For each category, the percentage of scored genotypes in the collection is reported. References [69,70,71,72,73,74,75,76,77,78] are cited in the Supplementary Materials.
