# Supplementary material for: Biodiversity Evaluation and Preservation of Italian Stone Fruit Germplasm (Peach and Apricot) in Southern Italy
Source: Plants (Basel). 2023 Mar 11;12(6):1279. doi: 10.3390/plants12061279 (PMC10055517; doi:10.3390/plants12061279)
Supplement: Supplementary file 1 [file plants-12-01279-s001.zip › plants-2221737-supplementary.pdf]

Table S1. List of morphological and phenological traits recorded in apricot and peach collections.

	Trait	Apricot	Peach
**Tree**	Habit	√	√
	Vigour	√	√
**One year old branch**	Colour	√	-
	Bud shape	√	-
	Bud size	√	-
	Apex anthocyanin colour	√	√
	Thickness	-	√
	Internode length	-	√
	Number of early branches	-	√
**Flower**	Size	-	√
	Petal colour	-	√
	Petal shape	-	√
**Leaf**	Size	√	√
	Margin	√	√
	Colour intensity of upper page	√	√
	Petiole length	√	-
	Petiole upper side colour	√	-
	Gland size	√	-
	Pedicular gland number	-	√
	Pedicular gland shape	-	√
**Fruit**	Size	√	√
	Symmetry	√	√
	Shape	√	-
	Shape of apex	√	-
	Prominence of suture	√	√
	Type of skin surface	√	-
	Skin ground colour	√	√
	Hue of skin over-colour	√	√
	Glossiness of skin over-colour	-	√
	Pattern of skin over-colour	-	√
	Skin thickness	-	√
	Skin adherence to flesh	-	√
	Flesh colour	√	√
	Flesh texture	√	√
	Flesh fiber	-	√
	Flesh firmness	√	√
	Flesh adherence to stone	√	√
	Aroma and taste	√	√
	Acidity	√	-
**Stone**	Size	√	√
	Shape	√	-
	Kernel bitterness	√	-

	Ratio to flesh	√	-
	Brown colour intensity	-	√
	Surface relief	-	√
**Phenology and productivity**	Flowering time	-	√
	Harvest maturity	√	√
	Productivity	√	√
	Fruit resistance to handling	√	√
Total		*33*	*36*

Table S2. List of microsatellites used to analyze apricot and peach collections. Locus type, species of origin (*Prunus* spp), repeat motif and annealing temperature (Ta in °C) are specified.

SSR locus	Type	Species	Motif	Ta	Reference	Apricot (*P. armenica*)	Peach (*P. persica*)
BPPCT001	SSR	*P. persica*	$(GA)_{27}$	57	[69]	√	√
BPPCT007	SSR	*P. persica*	$(AG)_{22}(CG)_{2}(AG)_{4}$	57	[69]	√	√
BPPCT010	SSR	*P. persica*	$(AG)_{4}GG(AG)_{10}$	57	[69]	√	-
BPPCT014	SSR	*P. persica*	$(AG)_{23}$	57	[69]	√	-
BPPCT015	SSR	*P. persica*	$(AG)_{13}$	57	[69]	-	√
BPPCT017	SSR	*P. persica*	$(GA)_{28}$	57	[69]	-	√
BPPCT025	SSR	*P. persica*	$(GA)_{29}$	57	[69]	√	√
BPPCT038	SSR	*P. persica*	$(GA)_{25}$	57	[69]	-	√
CPDCT025	SSR	*P. dulcis*	$(CT)_{10}$	62	[70]	√	-
CPDCT045	SSR	*P. dulcis*	$(GA)_{16}$	62	[71]	√	√
CPPCT006	SSR	*P. persica*	$(CT)_{16}$	59	[72]	√	√
CPPCT022	SSR	*P. persica*	$(CT)_{28}CAA(CT)_{20}$	50	[72]	-	√
CPPCT033	SSR	*P. persica*	$(CT)_{16}$	50	[72]	√	√
CPSCT012	SSR	*P. salicina*	GA	62	[70]	√	-
CPSCT018	SSR	*P. salicina*	$(CA)_{5}(CT)_{20}$	52	[70]	√	-
CPPCT044	SSR	*P. persica*	$(CT)_{22}$	58	[31]	-	√
CPSCT012	SSR	*P. salicina*	GA	62	[70]	-	√
EPPCU5176	EST-SSR	*P. persica*	-	55	[73]	-	√
PCHGMS1	SSR	*P. persica*	$(AC)_{12}(AT)_{6}$	60	[74]	√	-
UDP96003	SSR	*P. persica*	$(CT)_{11}(CA)_{28}$	57	[75]	√	-
UDP96005	SSR	*P. persica*	$(AC)_{16}TG(CT)_{2}CA(CT)_{11}$	57	[75]	-	√
UDP96008	SSR	*P. persica*	$(CA)_{23}$	50	[75]	-	√
UDP98022	SSR	*P. persica*	$(TG)_{12}(AG)_{24}$	64	[76]	-	√
UDP98409	SSR	*P. persica*	$(AG)_{19}$	57	[75]	√	√
UDP98412	SSR	*P. persica*	$(AG)_{28}$	57	[76]	√	√
Total						*15 SSR*	*18 SSR*

According to the Genome Database of *Rosaceae* (GDR) [77,78]

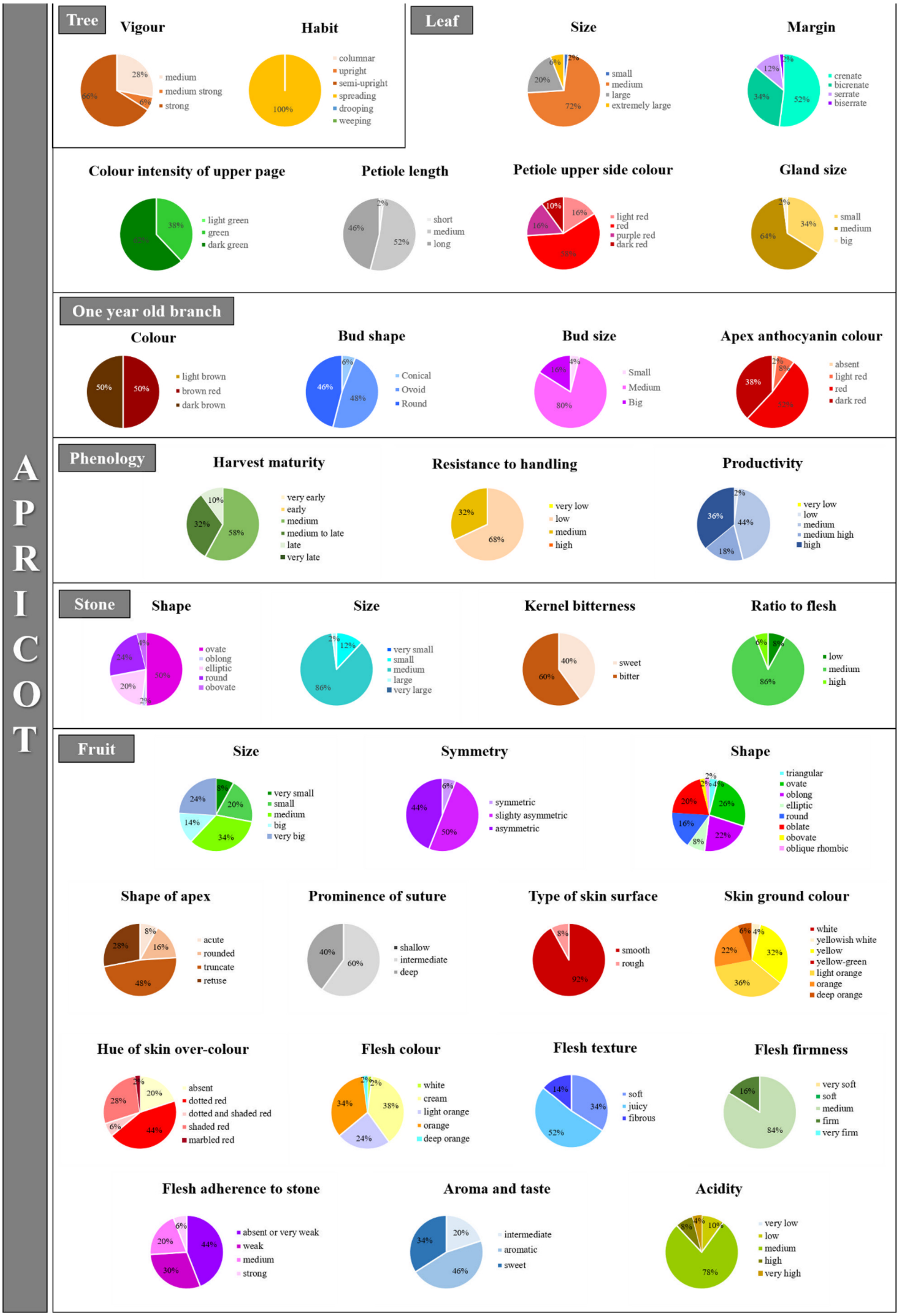

Figure S1. Graphical representation of apricot morphological and phenological categories detected for all the considered traits. For each category, the percentage of scored genotypes in the collection is reported.

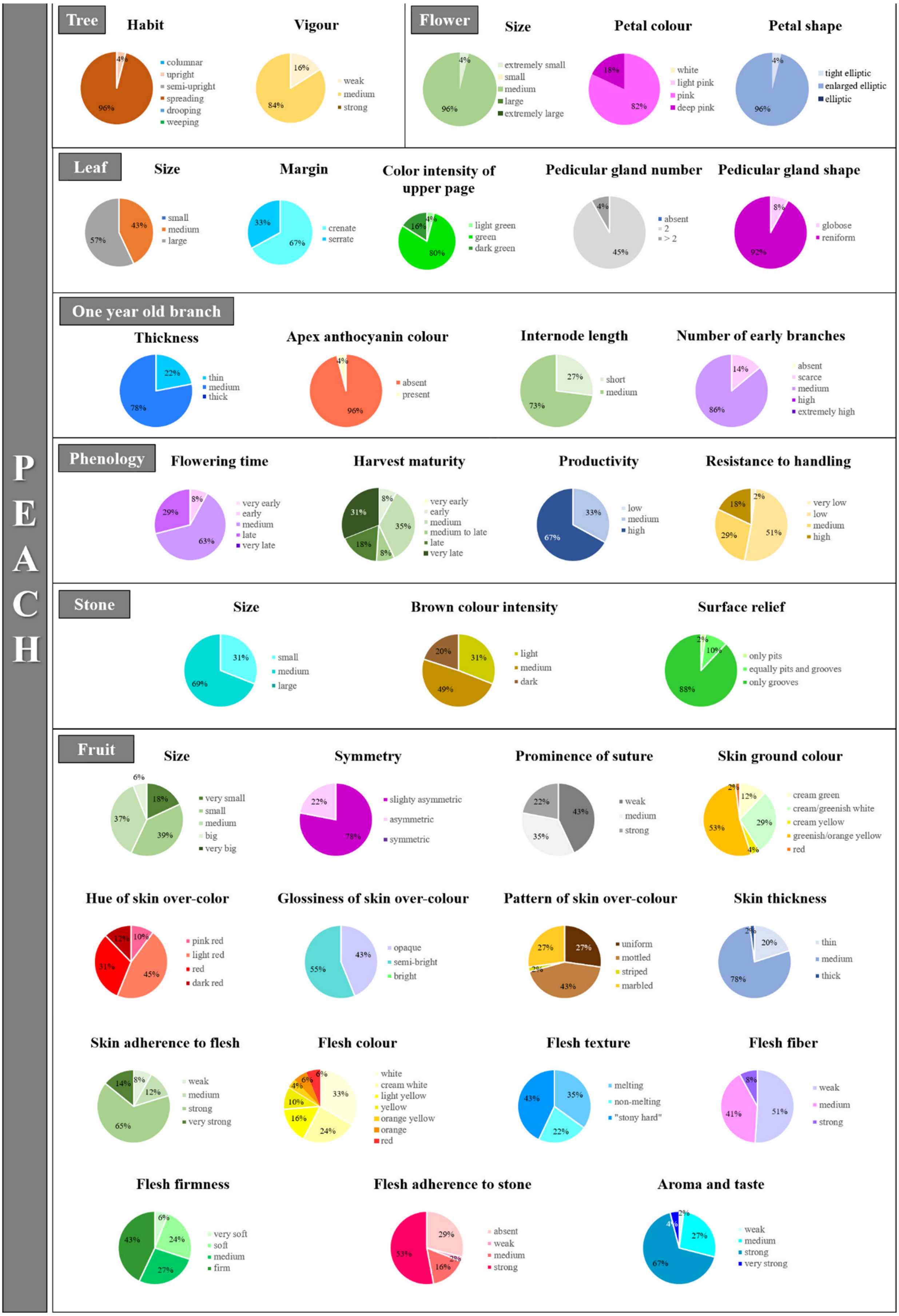

Figure S2. Graphical representation of peach morphological and phenological categories detected for all the considered traits. For each category, the percentage of scored genotypes in the collection is reported.
